# Double breasting repair of urethral fistula in a patient with perineal abscess secondary to a large urethral stone

**DOI:** 10.4103/0970-0358.44938

**Published:** 2008

**Authors:** Sankalp Dwivedi, S.R. Joharapurkar, Abhay Deshmukh

**Affiliations:** Department of Surgery, Datta Meghe Institute of Medical Sciences, Sawangi (Meghe), Wardha, India

**Keywords:** Double breasting, urethra, urethral fistula, urethral diverticulum

## Abstract

We report here a case of urethral fistula managed successfully following incision and drainage for the urethral abscess secondary to a large urethral stone leading to a large diverticulum (another rare condition) by using the technique of double breasting, where we used the redundant urethra and overlying skin.

## INTRODUCTION

Giant acquired diverticulum of the urethra is rare and the cause is usually a stricture of the penile part of the urethra.[1] We report here a case of urethral fistula managed successfully following incision and drainage for the urethral abscess secondary to a large urethral stone leading to a large diverticulum (another rare condition)[2] by using the technique of double breasting, where we used the redundant urethra and overlying skin.

## CASE REPORT

**Figure 1:**
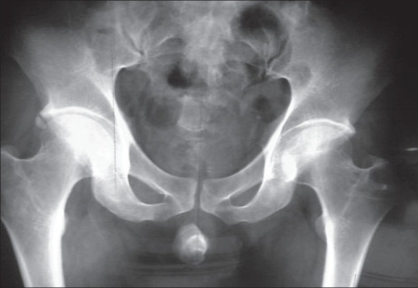
X-ray of the pelvis showing a large urethral stone

**Figure 2:**
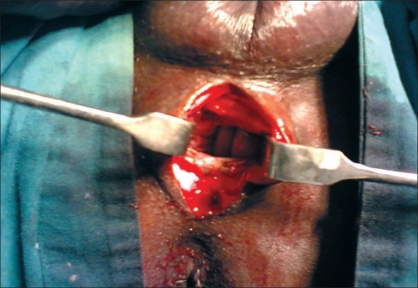
Large space following incision and drainage

**Figure 3:**
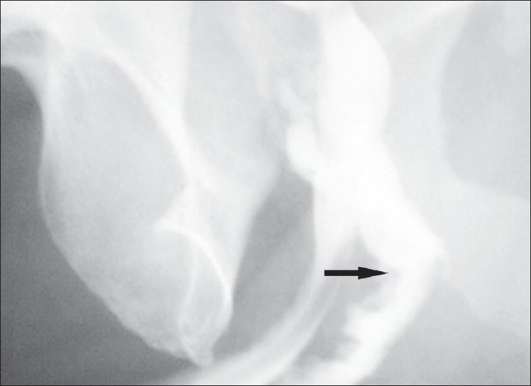
Preoperative urethrogram showing the site of leak (arrow)

**Figure 4:**
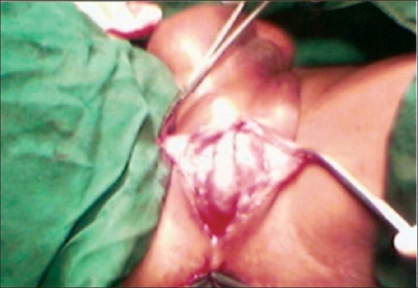
Redundant urethra after excision of the fistulous tract

**Figure 5:**
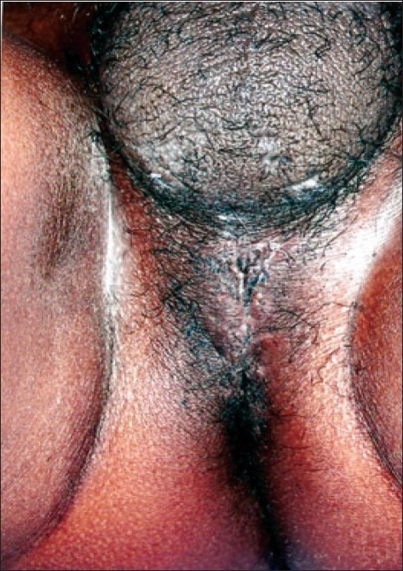
Well healed wound at follow-up

A 40 year-old male patient was treated in the emergency room for a large perineal abscess. Examination revealed that he had an approximately 9 × 9 cm^2^ tense, tender, fluctuating swelling with the features of acute inflammation in the perineum. The X-ray of the pelvic region showed a large stone in the bulbar urethra [Figure 1]. He underwent incision and drainage of the abscess and removal of the stone (8 × 5 cm^2^) in the emergency room [Figure 2]. He was postoperatively managed with daily dressing, and antibiotics were continued according to the results of cultures and sensitivity. He was discharged with the catheter *in situ* after 15 days but in the three months' follow-up visit, he complained of discharge of urine from the perineum. Local examination showed that there was an about 0.5 × 0.5 cm^2^ sized opening in the perineal region at the level of the bulbar urethra with continuous dribbling of urine. Urine examination showed 8–10 pus cells and growth of *Pseudomonas* and *E. coli* sensitive to Amikacin and Ceftriaxone was noted upon culture. He was treated with the above antibiotics until the urine culture became sterile. A fistulogram was taken to examine the fistulous tract, which was seen to be continuous with the bulbar urethra [Figure 3]. Surgical exploration and repair of the fistula were planned. There was healthy and adequate redundant bulbar urethra after excision of the fistulous tract. In contrast to the stricture cases, there was redundant urethral flap secondary to the presence of a large urethral stone in our case. We decided to use the technique of double breasting to repair and reinforce the urethra by utilizing this redundant urethral flap as it would provide an adequate urethral lumen and lax suture line after stripping the urethral mucosa of the redundant flap [Figure 4]. This was further reinforced with reimposition of corpus spongiosa. The wound was closed in layers and healed well. Sutures were removed on the 10^th^ day and the urethral catheter was removed on the 18^th^ day [Figure 5]. The patient was seen to have good force and stream of urine without any leakage. Further investigations to rule out stricture of the urethra did not reveal any abnormalities. The patient is on regular follow-up and doing well three months after surgery.

## DISCUSSION

While repairing urethral strictures, several techniques have been used to prevent fistula formation with good success rates. These include urethral covering by a well-vascularised, dorsal, double-layer dartos flap,[3] application of a tunica vaginalis flap,[4] and buccal mucosa grafting.[5] In one report, the buccal mucosa was further reinforced with a genital skin flap and this reduced local as well as systemic complications.[6] We combined these time-tested techniques in our patient and we used redundant urethral mucosa for primary repair in a double breasting manner and reinforced it by corpus spongiosa fibres. The perineal skin was closed over it. In conclusion, with our method of surgical treatment and with postoperative care, the patient is doing well at follow-up.
